# Annotations

**Published:** 1908-10-31

**Authors:** 


### Annotations.

### The late Professor Paul Berger.

The tragic death of Professor Paul Berger removes one of the best known and most widely respected of the members of the medical faculty of Paris. As a surgeon he won a reputation as a clever operator and an expert diagnostician, and by his writings and contributions to medical and surgical literature he gained a niche for himself in the large temple of medical fame, where so many of his fellow-countrymen have entered as laureates. To English students who came to Paris to study his methods he was always the pink of considerateness and kindness, and there were few audiences so cosmopolitan as those which he addressed at the Hospital Necker. As an operator his expertness was astonishing, although he had almost an old-world sedateness in his methods. He took a keen interest in the advance of surgery, especially in that part of it which he had made his speciality—abdominal operations and the radical cure of hernia. As a teacher he was precise and systematic, illustrating his remarks from an interesting, rich, and varied series of cases. He gained the highest honours in his profession, and remained on the staff of his old hospital, dying literally in harness, as he was struck down by apoplexy with the scalpel in his hand just as he was about to operate on a case. His death deprives English and American post-graduate students of one of their best friends and most courteous supporters among the professors of the French faculty.

### Fads and Fallacies in Child Management.

At the Infants' Health Exhibition, which has been held during the last fortnight at the Institute of Hygiene, a course of popular lectures has been delivered on the natural history and hygiene of infancy by medical men and women who have made special study of these subjects, and it is probable that the principles laid down by them may have some good effect upon the public mind. The problems of infant feeding and child management are much obscured by popular customs and superstitions, and there is therefore ample scope for the popular exposition of more enlightened views and principles. Dr. Francis Warner criticised present-day methods of child education. He especially advocated eye-training as the best method of impressing the minds of children and teaching them to acquire useful methods of thought. Dr. Warner also condemned all constriction of the fingers and toes of infants, on the grounds that this checking of the spontaneous movements of small parts hinders the early development of the brain, of which they are the objective manifestations. Dr. Lilian Chesney's lecture on October 22 was largely an appeal for common sense in the management of children in health and disease. She spoke of the importance of fresh air during the long hours of bedtime in childhood, and of the necessity of a cleanly skin for cutaneous transpiration. Russian tallow, camphorated oil, and excessive clothing, so dear to the hearts of the mothers of the hospital out-patient class, were all vigorously condemned. Dr. Chesney has a turn for incisive and epigrammatical expression, and she uses this with considerable effect against the fads and superstitions which disfigure the upbringing of infants in all classes of society. Although there was little that was new and nothing that was revolutionary in her address, Dr. Chesney succeeded in putting doctrines which are well accepted by the medical profession into language which was likely to impress the non-medical mind. The apt exponent of hygienic truths in popular terms will probably have a definite place in the public health service of the future, for it is certain that simple and pointed phraseology is a factor of great importance in the dissemination of sound principles. The short and simple text, "Do not spit," which is now so liberally plastered over public vehicles and meeting places, is perhaps a greater hindrance to the spread of tuberculosis than any of the other more complicated methods of educating the public in this respect.

### Employment versus Lactation.

For a number of reasons many women of the labouring class are compelled to wean their infants very soon after birth, and to resume arduous occupations at a much earlier date after delivery than is desirable in the interests of their own health. From this arise baby farms, hand-feeding, and other causes of infantile mortality, and debilitating conditions of many kinds such as can be found crowding the out-patient rooms of women's hospitals. The Research Committee of the Christian Social Union has conducted an inquiry into the employment of mothers after childbirth, to ascertain the facts of the matter statistically, and their recently published report discloses some very interesting points. Thus of women whose employment consisted of household duties only, 433 out of 638 worked up to the time of their confinement, and 338 resumed their duties between the tenth and fourteenth days. The infantile death-rate among the children was 126.9 per 1,000; this was much lower than with the women whose work was industrial, whether performed at home or away, among whose families it reached 194.2. This difference is only to be expected when it is borne in mind that the proportion of breast-fed infants is higher among the first class, and that careful hand-feeding is also much easier to ensure than for the industrial workers. The contrast between the results of maternal nursing and artificial feeding is also well pointed by the mortality figures of the whole series, which are for the former 145 per 1,000, against 230 per 1,000 for the latter. It appears, however, that even of the mothers who have to go out to work some 60 per cent. manage to nurse their children, and 20 per cent. more do so in part. It is evident, therefore, that artificial feeding cannot be entirely to blame for the higher death-rate among these mothers' children, and there can be no doubt that work of this sort, especially when resumed very soon after delivery, is a source of peril both by conducing to irregular times of nursing and by deteriorating the health of the mother and the quality of her milk.

